# Antiretroviral therapy supply chain quality control and assurance in improving people living with HIV therapeutic outcomes in Cameroon

**DOI:** 10.1186/s12981-017-0147-x

**Published:** 2017-04-04

**Authors:** M. P. Ngogang Djobet, David Singhe, Julienne Lohoue, Christopher Kuaban, Jeanne Ngogang, Ernest Tambo

**Affiliations:** 1 LABOREB—Laboratoire de Recherche et d’Expertise Biomédicale, Yaoundé, Cameroon; 2 LANACOME, National Laboratory for Drugs Quality Control, Ministry of Public Health, Yaoundé, Cameroon; 3 Faculty of Medicine and Biomedical Sciences, University of Yaoundé I, Yaoundé, Cameroon; 4 Faculty of Medicine, University of Bamenda, Bamenda, Cameroon; 5 Université des Montagnes, Bangangté, Cameroon; 6 Africa Disease Intelligence and Surveillance, Communication and Response (Africa DISCoR) Institute, Yaoundé, Cameroon

**Keywords:** Quality, ARVs, HIV/AIDS, Therapeutic, Outcome, Medicine, Drugs

## Abstract

**Background:**

Evaluation of medication efficacy and safety is an essential guarantee to successful therapeutic outcome in public health practices. However, larger distribution chain supply in developing countries such as Cameroon is often challenged by counterfeit drugs, poor manufacturing, storage and degradation leading to health and patient adverse consequences. Yet, access to supply chain management in strengthening ARVs quality assurance and outcomes remains poorly documented. More than 53,000 patients have been enrolled on free ARVs medications, but little is documented on quality assurance and validity of safety for affected populations along the supply chain management since 2008.

**Methods:**

The cross sectional study was conducted in ARVs distribution units and centers in central, littoral and south west regions of Cameroon. ARVs drugs samples included Nevirapine, Efavirenz, and fixed dose combinations of Zidovudine + Lamivudine, Lamivudine + Stavudine and Zidovudine + Lamivudine + Nevirapine. Drugs packaging and labeling was assessed and galenic assays were performed at National Laboratory of quality Control of Medications and Expertise (LANACOME), Yaoundé, Cameroon.

**Results:**

The study covered 16 structures located in eight different towns including the central ARVs store, two regional pharmaceutical procurement centers and thirteen HIV approved treatment centers and management units. A total of 35 ARVs products were collected. Only eight ARVs drugs containing Lamivudine and Stavudine presented with white stains on tablets, however these drugs were standard for all other tests performed. The others 28 ARVs products were standards to all assays performed.

**Conclusion:**

We concluded that ARVs drugs freely accessible and distributed to PLWHA are of good quality in Cameroon. However, with the increase number of patients under HAART since 2013, adoption of “Test and Treat” approach to reach the 90-90-90 goals and with the implementation of new national antiretroviral regimen guidelines and molecules such as boosted protease inhibitors, continuous quality control and assurance surveillance, monitoring and evaluation is recommended. Assessment of quality of formulations that are more susceptible to degradation such as pediatric formulations for averting the rising multidrug resistance trend is also desired.

## Background

Although scaling up of antiretroviral therapy (ART) is clearly documented across epidemics countries in Africa and elsewhere, ARVs supply chain quality control and assurance in terms of conformity, content, dosage as well as packaging and labeling instructions on use in most developing countries, and Africa in particular is still poorly understood [1–3]. Yet, it is also documented that counterfeit drugs, poor manufacturing and unregulated drugs are found or reported in most developing countries including Cameroon [4]. This situation is worsened by multifactorial challenges and issues including the lack of competent local regulatory authorities, lack or inexistent drugs quality control laboratories, and poverty related socio-economic factors [6–8]. These gaps explain why sub standards medications may also be found in legal drug supply chain [9, 10].

Prior findings have raised concern on presence of substandard antiretroviral drugs in some countries in Africa [11–14]. Sustained access to and use of such poor quality antimicrobial drugs may result in harmful consequences such as serious adverse effects impacting on patient’s adherence as well as therapeutic failure and antimicrobial resistance rising disability, morbidity and mortality [15, 16]. While on the other hand, the effective use and compliance to highly active antiretroviral therapy (HAART) and HIV service delivery have shown to significantly reduce morbidity and mortality in people living with HIV/AIDS (PLHA) by preventing relapse and opportunistic infections as well as improvement of quality of life and productivity of patients [17–19]. In Cameroon, data and information related to ARVs quality is based on a 2005 joint project in seven African countries [20] with the adoption and implementation of free ARVs treatment since 2008, thousands of people living with HIV/AIDS (PLWHA) are under HAARVs in the country.

The study aimed at evaluating the quality control and assurance of antiretroviral (ARVs) drugs supply chain and distribution in order to improve therapeutic service delivery and outcomes in PLWHA in Cameroon.

## Methods

### Study site and population

In Cameroon, ARVs drugs are freely distributed in HIV approved treatment centers (ATC) and management units (MU) which are directly supplied by corresponding regional pharmaceutical procurement centers (RPPC). All regional procurement centers (10 all over the country) are supplied by unique Essential Drugs National Procurement Center (EDNPC). In this study antiretroviral drugs were collected in Central, Littoral and South West regions reported to have higher rates of follow up of HAART patients. In each region, five approved treatment centers or management units were randomly selected in both urban and semi-urban settings. The study lasted for 7 months from 2008 to Jan 2009.

### Ethical clearance

The ethical authorization and clearance was obtained from the National AIDS Control Committee (NACC) committee and the Ministry of Health, Cameroon. All samples analyses were performed in LANACOME, Yaoundé Cameroon.

### ARVs selection criteria and collection

Antiretroviral active principle selected were those frequently prescribed and consumed within the country as first line regimen for adults [21]. These included the following active principle: Efavirenz, Nevirapine and fixed dose combination containing Lamivudine + Zidovudine, Lamivudine + Stavudine or Zidovudine + Lamivudine + Nevirapine. An administrative authorization from the Ministry of Public Health allowed collection of one or a maximum of two boxes of medications containing the selected active principle within the selected facilities. ARVs medications were provided depending on the stocks available in each facility. A code containing the generic name of the drug as well as the site and date of collection was assigned to each medication box. Boxes were placed into a sterile sealed plastic bag with suitable conditioning and transported to LANACOME laboratory for further analysis.

### Inspection of ARVs, packaging characteristics

Each medication box was inspected prior analysis to describe packaging characteristics. Primary and secondary containers and labeling information were verified namely: product’s brand name, active principle(s) name(s) and quantity, number of tablets, manufacturer’s reference number ,date of manufacturing and expiration, storage conditions, as well as manufacturer’s or retailer address. Tablet’s color uniformity, odor and presence or absence of powder at the bottom of the secondary container was also recorded on a sheet designed for the purpose of the study.

### Determination of tablet weight uniformity, hardness and disintegration time

The determination of weight uniformity of each sample was done using 20 tablets randomly selected inside the secondary container. Analytical precision balance at 0.01 g METTLER PM 400^®^ auto calibrated was used to weight serially one table after another and weight were recorded. The average weight was calculated as well as the standard deviation. Hardness testing was performed on 10 tablets using ERWEKA^®^ TBH 28 and the crushing power of each tablet was expressed in mean Kp. Disintegration time was defined using Pharma test^®^ PT2 Auto 3. Here six tablets of each ARVs was dropped in an apparatus containing distilled water at room temperature and the complete disintegration time was followed up over 15 min and/or repeated in case of failure at time lapsed using (6-6-6 = 18) tablets.

### Identification and evaluation of ARVs quality and concentration

ARVs identification and dosage tests were based on European an American Pharmacopeia and all ARVs products analysis were conducted in LANACOME, Yaoundé, Cameroon.

For the purpose of identification of ARVs, thin layer chromatography (TLC) was used to identify each given ARVs active principle using silica gel F245 with 95% ethanol diluting solvent. The development solvent was composed by mixed n-Butanol (40 ml), cyclohexane (30 ml), acetone (30 ml) and ammonia (10 ml). For preparation of standards, 10 mg of each standard was mixed with 10 ml of diluting solvent and homogenized using a vortex. For drug samples, one tablet of drug sample was mixed with 10 ml diluting solvent and homogenized. 5 µl of each standard and sample solutions where placed at 2 cm from the edge of the plate which was then placed inside the chromatography box saturated with the development solvent. After 15 cm of migration, the migration plate was removed and dried at room temperature. Plate was revealed under 254 nm UV light. Identification was considered conform if samples migrated at the same position than standards. Position of migration for each active principle was drawn on an overlay sheet to keep as record.

For concentration (dosage) of active principle(s), it was done through spectrophotometry analyses based on American Pharmacopeia in line with manufacturers labeling specifications. For Nevirapine and Efavirenz samples, tablets were crushed and a quantity corresponding to half a tablet was introduced into a 50 ml graduated flask and then 95% ethanol was added. This S1 solution was sonicated and further diluted into a 1:25 ratio solution (S2) that was also sonicated. Absorbance of S2 solution was measured around 287 nm for Nevirapine and 265 nm for Efavirenz. Drug dosage content was calculated using the following formula:

C = (Standard specimen × optical density of sample × dilution rate of sample/dilution rate of standard × optical density of standard × sample specimen) × mean weight of the tablet in mg.

Drug content was expressed in mg.

For fixed dose combinations, standard were prepared following the steps stated above. For FDC samples, half a tablet of drugs was dissolved in 250 ml of diluted hydrochloric acid (1:62): S1 solution. This S1 solution was diluted into a 1:10 S2 solution that was sonicated again for 15 min. Absorbance of solutions was measured around 263 nm for Stavudine, 265 nm for Zidovudine and 280 nm for Lamivudine.

### Data analysis

All data were reported, processed and analyzed on Microsoft office, Excel spreadsheet (version 2007). Results of the conformity and quality and labeling of ARVs drugs tested were expressed in percentage.

## Results

### Classification of active principles per structures

The study covered 16 structures of the supply chain distribution and storage in eight different towns, including the Essential Drugs National Procurement Center (EDNPC): Central level, two regional pharmaceutical procurement centers (RPPC): Intermediate level and 13 treatment centers: peripheral level including five approved treatment centers (ATC) and 08 management units (MU). The antiretroviral drugs collected were all generics. We assessed the quality of 35 ARVs drugs. From the 35 ARVs drugs collected, four were collected at the central level of the supply chain, seven at the intermediate level and 24 at peripheral level. 19 ARVs drugs were fixed dose combination. Table 1 summarized the distribution of active principle collected per structure (Table 1).

**Table 1:** Classification of active principle (s) per local structure in the three regions

Name of active(s) ingredient(s)	Total numbers of samples	Structures			
		Central level (EDNPC)	Intermediate level (RPPC)	Peripheral level (ATC) (MU)	
Efavirenz tablet 200 mg	2	1	–	1	–
Efavirenz tablet 600 mg	7	–	2	2	3
Lamivudine 150 mg + Stavudine 30 mg tablets	9	1	2	2	4
Lamivudine 150 mg + Zidovudine 300 mg tablets	7	1	1	2	3
Nevirapine 200 mg tablets	7	1	1	2	3
Zidovudine 300 mg + Lamivudine 150 mg + Nevirapine 200 mg	3	–	1	–	2
Total	35	4	7	9	15

### Assessment of packaging and labeling characteristics for collected ARVs

All the 35 collected ARVs were packed in two containers (primary and secondary container). None of these drugs had the expiration date due. All the tablets were stored in bottles (primary containers) made with vinyl-polychloride and 80% of these bottles were sealed on top with aluminium foil. More than half (64%) of these bottles had both hydrophilic cotton, aluminium foil and a dryer. All tablets had a homogeneous colour and normal odour, except those of eight (8) Lamivudine + Stavudine fixed dose combination from the same manufacturer whose pink tablets presented with white stains. None of the boxes had broken tablets. All required information was available on the primary container leaflets or patients instructions.

### Assessment of tablets hardness/shelf life, weight, and disintegration time

There was uniformity of weight for tablets for all these drugs. Friability and hardness testing as well as disintegration or residual degradation time was also conforming to general WHO pharmacopoeia standards.

### Identification of active ingredient(s)/principle(s)

**Fig. 1:**
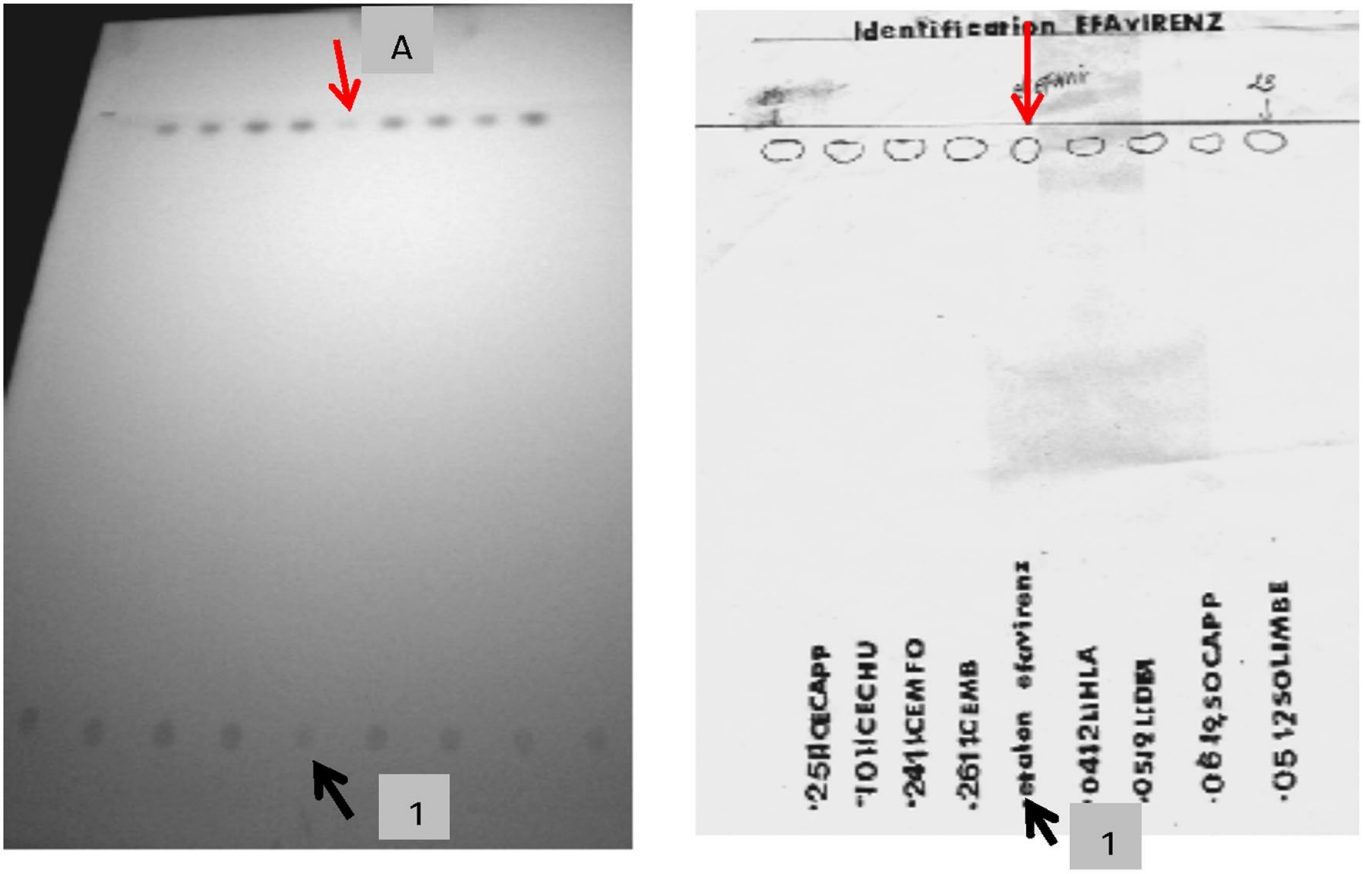
TLC for Efavirenz samples, plate reveal under ultraviolet (UV) light. * 1* Deposit site of standards and samples. * A* Migration site for efavirenz standard

**Fig. 2:**
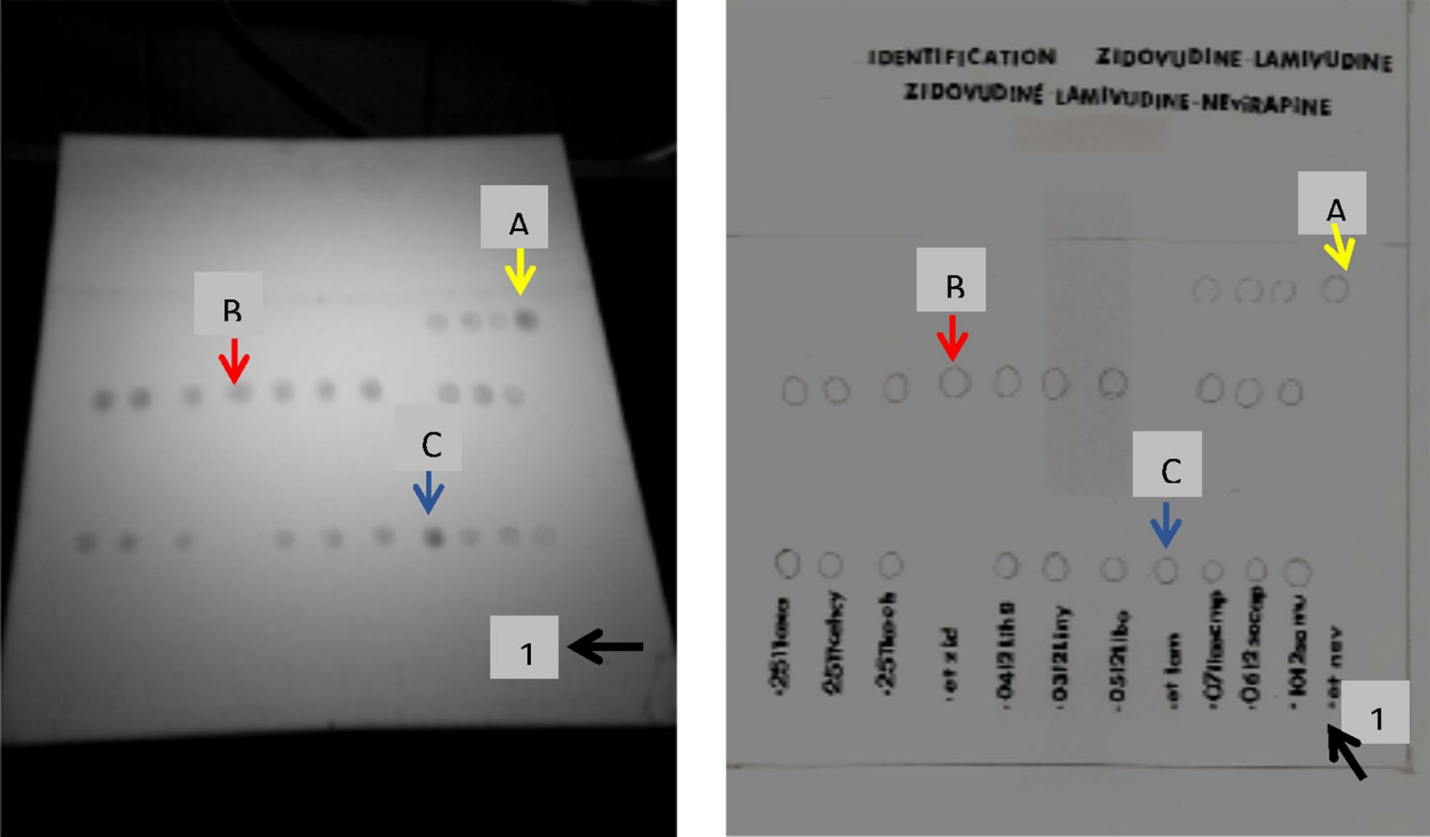
TLC for fixed dose combinations containing Zidovudine, Lamivudine and Zidovudine, Lamivudine and Nevirapine, plate reveal under UV light. *1* Deposit site of standards and samples. *A* Migration site for nevirapine standard. *B* Migration site for zidovudine standard. *C* Migration site for lamivudine standard

Identification of active principle was performed using thin layer chromatography (TLC). Identification was in concordance for all the collected ARVs. For example, the identification of Efavirenz and fixed dose combination tablets containing Zidovudine, Lamivudine and Nevirapine is shown in Figs. 1 and 2.

In Fig. 1, the image shows on the left, the plate reveal under UV light for eight Efavirenz samples. Image on the right shows overlay sheets of the same plate. The standard position is indicated with the red arrow. All samples tested migrated at the same position as for the standard. There weren’t additional spots along the migration way thus assuming purity of these medications (Fig. 1).

In Fig. 2, the image shows identification of active principle in six fixed dose combination containing Zidovudine and Lamivudine and three fixed dose combination containing Zidovudine, Lamivudine and Nevirapine. The black arrow indicates the position where samples were deposited. Red arrow indicates position of migration of Zidovudine standard, while blue and yellow arrows indicate position of lamivudine and Nevirapine standards respectively. Identification was in concordance for these nine fixed dose combinations. No additional spots were observed during this migration thus assuming purity of these medications (Fig. 2).

### Assessment of the concentration of active principle per tablet

**Fig. 3:**
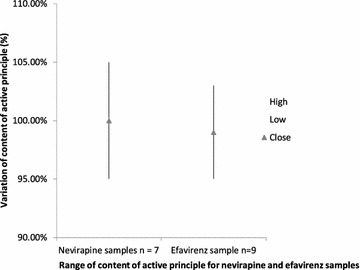
Range of active principle concentration for Nevirapine and Efavirenz

According to American’s pharmacopeia, concentration of active principles should range between 90 and 110% of the dosage stated on the container. This range was respected for the 35 ARVs collected. Figure 3 shows range of content for Nevirapine and Efavirenz samples. Dosage of active principle in Nevirapine samples varied between 95.85 and 105.36% of the stated dose while for Efavirenz tablets, variation was between 95.9 and 103% (Fig. 3).

## Discussions

The study assessed the quality of 35 ARVs drugs collected; four were collected at the central level of the supply chain, seven at the intermediate level and 24 at peripheral level. 19 ARVs drugs were fixed dose combination. It also provided insights on ARVs supply chain adherence in Cameroon. It showed that ARVs tablets weight were uniform for all these drugs, while the hardness (friability) and hardness testing as well as disintegration time were also conforming. None of these drugs had the expiration date due. As packaging preserves the stability and quality of medicinal products, packaging of ARVs collected was primarily assessed using WHO recommendations [23]. There was proper labelling for all the medication boxes collected and specific description of containers revealed that all the tablets were stored in bottles (primary containers) made with vinyl-polychloride and 80% of these bottles were sealed on top with aluminium foil. More than half (64%) of these bottles had both hydrophilic cotton, aluminium foil and a dryer. Sealing with aluminium foil and combination of cotton, aluminium foil and dryer are elements of the manufacturing process that contribute to enhance drug stability. All tablets had a homogeneous colour and normal odour, except those of eight (8) Lamivudine + Stavudine fixed dose combination from the same manufacturer whose pink tablets presented with white stains. None of the boxes had broken tablets. Regarding classification of principles and structure, the selection of ARVs was based on rate of consumption of antiretroviral drugs obtained from the National Aids Control Committee report [21]. Only active principle from first line regimen were considered (nucleosidic and non nucleosidic inhibitors) because of their frequent use and also because by that time, not many people were under second line regimen including protease inhibitors. Since drugs are freely distributed, stocks of medications are strictly calculated for each region, management units and treatment centers from central level of the supply chain. Thus we were given ARVs drugs in such a way not to disrupt stocks in each structure and active principle depended on availability. This explains the variability in the number and the nature of active principle collected among the various structures. We could not obtain drugs from one of the structure at the intermediate level, because of additional administrative procedures requested in that structure.

Our findings from the assessment of formulation, labeling and packaging characteristics of different collected ARVs revealed that all these ARVs were properly packed according to WHO guidelines on packaging for pharmaceutical products [22]. Appropriate packaging of the generic drugs can be explained by the fact that all the medications were WHO prequalified products, meaning they were manufactured in sites audited and recognized by WHO to respect quality assurance during manufacturing process [23]. Use of prequalified drugs has been set up by the government to ensure good quality of ARVs available in the country.

During inspection procedure, Lamivudine and Stavudine fixed dose combination tablets from the same pharmaceutical company did not have a homogeneous colour. This lack of uniformity was observed on tablets of all the samples collected in the various regions. However, they conformed to all others tests performed, suggesting that this lack of uniformity of colour may be related to manufacturing process and was not a result of tablet’s degradation. Nonetheless, Stavudine regimen have been progressively phased out in the country [24] and are not anymore used due to HIV patients severe toxicity widely reported [25, 26].

Findings from assessment of physico–chemical properties, identification and active principle dosage showed that all the drugs collected complied for the tests performed. Good quality of medications recorded could firstly be explained by the fact that procurement process for these free ARVs drugs in the country follow up strong regulations and secondly all brands collected were WHO prequalified products [23], guaranteeing the source of manufacturing. Our findings were consistent with previous assessment of antiretroviral drugs quality in seven African countries where Cameroon was the second country using pre-qualified drugs (68% of antiretroviral available) compared to Uganda, Nigeria and Zambia with respectively 32.33 and 34% of use [20].

Methods used for identification and active principle dosage in this study were those available at LANACOME. Although there are more sensitive methods such as high liquid performance chromatography that could be used [27], thin layer chromatography and spectrometry analyses have been demonstrated to remain good practices for identification and dosage of drugs, especially in country where accessibility to expensive equipment as for high performance liquid chromatography (HPLC) is not possible [28]. Baseline characterization of active principle(s) concentration per tablet and per class of ARV drugs were consistent with standard recommended by WHO guidelines and manufacturers labels/patients instructions. Good quality of antiretroviral drugs found in our study was consistent with other studies [29–31]. However, other relevant issues that rose from previous findings in other studies such as storage conditions and their impact on quality of medications were not documented [2–5, 32–34].

The findings limitations included limited access to other regions of Cameroon in assessing the quality of ARVs drugs, only tablets formulations could be tested and lack of equipment and facilities for liquid formulations assessment at LANACOME.

## Conclusion

It was evident, ARVs supply chain distribution in terms of quality and conformity standards were consistent with international regulations in the three assessed regions. According to our findings, procurement of prequalified drugs is highly recommended to avoid distribution of fake medication, especially for ARVs, since manufacturing sites for these drugs are numerous. Also, conducting other assessment studies in different settings is advisable to gather insights that can help to ensure safe, efficient and effective use of safe ARVs. Importantly, coherent and coordinated supply chain harmonization and coordination as well as continuous quality control monitoring and evaluation of all forms of drugs seems to be crucial in maintaining efficacy and safety towards PLWHA patients’ successful therapeutic care delivery and outcomes.

## Abbreviations

ARVs: antiretroviral
ATC: approved treatment center
EDNPC: Essential Drugs National Procurement Center
FDC: fixed dose combination
HPLC: high performance liquide chromatography
LANACOME: Laboratoire Nationale de Contrôle de Qualité des Médicaments et d’Expertise
MU: management unit
NACC: National AIDS Control Committee
PLWHA: people living with HIV/AIDS
RPPC: regional pharmaceutical procurement center
TLC: thin layer chromatography
WHO: World Health Organization
